# Microbiome changes: an indicator of Parkinson’s disease?

**DOI:** 10.1186/s40035-019-0175-7

**Published:** 2019-12-24

**Authors:** Caroline Haikal, Qian-Qian Chen, Jia-Yi Li

**Affiliations:** 1 Neural Plasticity and Repair Unit, Wallenberg Neuroscience Center, Department of Experimental Medical Science, BMC A10, 221 84 Lund, Sweden; 2 Institute of Neuroscience, College of Life and Health Sciences, Northeastern University, Shenyang, Liaoning China; 3 Institute of Health Sciences, China Medical University, Shenyang, 110112 China

**Keywords:** Parkinson’s disease, Intestinal microbiota, Inflammation, Gut, Protein aggregation

## Abstract

Parkinson’s disease is characterized by dopaminergic neuron loss and intracellular inclusions composed mainly of alpha synuclein (α-syn), but the mechanism of pathogenesis is still obscure. In recent years, more attention has been given to the gut as a key player in the initiation and progression of PD pathology. Several studies characterizing changes in the microbiome, particularly the gut microbiome, have been conducted. Although many studies found a decrease in the bacterial family Prevotellaceae and in butyrate-producing bacterial genera such as Roseburia and Faecalibacteria, and an increase in the genera Akkermansia many of the studies reported contradictory findings. In this review, we highlight the findings from the different studies and reflect on the future of microbiome studies in PD research.

## Background

### Introduction

With diseases where genetic factors and identified environmental causes account only for a minority of the cases, unidentified environmental factors are believed to play a key role in the initiation and progression of the disease. Such is the case with Parkinson’s disease (PD) wherein 90% of cases are of idiopathic nature [1]. In PD, dopaminergic neurons of the substantia nigra pars compacta degenerate and the protein alpha-synuclein (α-syn) misfolds and aggregates into Lewy bodies (LBs) and Lewy neurites (LNs). As Braak identified that α-syn inclusions appear in the dorsal motor nucleus of the vagus (DMNV) at very early stages of PD, he postulated that the pathology could be initiated in the gut or nose [1, 2]. Additionally, direct evidence of α-syn pathology propagating from the GI tract to the DMNV via the vagal nerve has been provided by two different studies in rodents [3, 4]. There is also a general consensus that a full truncal vagotomy decreases the risk of developing PD later in life [5, 6]. Moreover, up to 80% of PD patients experience constipation and delayed gastric emptying, often many years before motor symptoms appear [7]. To date, it is still of debate whether PD patients are at a higher risk of developing IBD [8–11]. A recent study showed that intestinal infection could trigger PD-like symptoms in genetically predisposed mice [12]. Other studies, which identified α-syn inclusions in intestinal biopsies years prior to PD diagnosis [13], have provided further evidence for this hypothesis. The gut, which harbors a large portion of our microbiome, acts as an intermediary between us and the environment and also plays pivotal roles in the priming and development of our immune system. That the microbiota in the GI system would play a role in synucleinopathies is supported by findings of bacterial substances modulating α-syn aggregation: both lipopolysaccharide [14, 15] and the different subunits of the *E. coli* amyloid protein Curli [16–18] have been shown to alter α-syn aggregation kinetics and produce fibrils with distinct toxicities compared to ‘pure’ fibrils. What concerns the intestinal microbiome, several studies have shown that germ-free mice display attenuated pathology and behavioral deficits in several models [19]. In a landmark study, Sampson et al. demonstrated that the microbiota itself could trigger or delay motor symptom onset in mice: Thy-1 α-syn mice colonized with microbiota from PD patients showed enhanced α-syn pathology load and microglial activation compared to mice colonized with microbiota from healthy individuals. Of note is that the study also found short chain fatty acids (SCFAs), metabolites of certain bacteria, to be sufficient in inducing α-syn pathology and microglial activation in the α-syn-overexpressing mice [19]. Of interest is that inflammation is a central component of PD pathology. Treatment with immunosuppressants has been shown to ameliorate PD pathology in mouse models [20] and mice lacking CD4+ T-cells have an attenuated response to MPTP with markedly decreased dopaminergic neuronal loss [21]. As emerging studies have found links between microbiome alterations, particularly gut dysbiosis, and a myriad of inflammation-driven diseases, including but not limited to inflammatory bowel disease (IBD), arthritis, liver disease, and obesity [22], it should come as no surprise that recent studies have aimed to identify changes in the microbiome of PD patients compared to healthy controls. In this review, we will summarize the findings from mostly human studies but also a few studies in animal models, discuss reasons behind the often contradictory findings, and relate the findings to studies in other disease conditions.

### The microbiome

Even though most studies have centered on the gut, the microbiome is not limited to the gastrointestinal (GI) system but rather also includes the microbiota in the nose epithelium, the skin, genitals, all mucosal surfaces and any other number of tissues naturally inhabited by microorganisms. Distinct body parts show distinct microbiomes. The development of the microbiome begins in utero although the actual birth marks the first major colonization of the child with studies showing marked differences in the gut microbiome in children born vaginally compared to those born via caesarean section (C-section) [23]. The microbiome of children born vaginally is characterized by Prevotella and Lactobacillus taxa whereas that of children born via C-section is dominated by Staphylococcus and Streptococcus species [24]. Children born via C-section more often carry Kliebsella and Enterobacter [25]. Additionally, colonization with Bifidobacteria and Bacteroides is delayed in children born via C-section [25–27]. These differences seem to have effects persisting into adulthood with children born vaginally being at lower risk of developing allergies, asthma, IBD and obesity compared to those born via C-section [23]. During the first 3 years of life, the microbiome is constantly changing but stabilizes afterwards and has been shown to be relatively constant throughout life. A study of monozygotic and dizygotic twins reported heritability of members of the Ruminococcaceae and Lachnospiraceae families and also of methanogens from the archaeal domain. Contrarily, Bacteroidetes were found to be mostly environmentally determined [28]. Factors such as lifestyle, diet and antibiotic use play considerable roles in shaping the microbiome [22]. Antibiotic usage has been shown in several studies, to both acutely and permanently perturb the gut microbiome, with a loss of diversity and a shift in community composition [29–31]. Long-lasting perturbations to the gut microbiome are also caused by infections with the pathogen *Clostridium difficile* [32]. It seems that ageing, too, affects gut microbial composition. Several studies have now shown that ageing in healthy adults is correlated with an increase in the diversity of microbial species, known as alpha-diversity [33]. Although there have been differences in the microbial species examined and the results reported, most studies have found Bifidobacteria and certain Firmicutes to be decreased in stool from healthy aged adults compared with younger subjects [33–35]. Finally, although it is difficult to assess whether the differences reported reflect the true situation within the intestines or is an artifact from sample collection, a fast transmission time has been correlated with a decrease in microbial diversity and an abundance of fast growing species and mucosal renewal [36–38].

### Main text

#### The microbiome in PD

Several studies have now aimed at identifying microbiota differences in PD patients compared to healthy controls. While most of these have used fecal samples as a proxy for the microbiota composition in the distal colon, some have examined mucosal biopsies or even salivary and nasal mucosa samples [39–41]. A summary of the findings from stool microbiome studies in PD patients can be found in Table 1 and a more comprehensive compilation of the findings in Additional file 1: Table S1. While only a few of the studies found significant differences in the alpha-diversity, that is the community richness and evenness within a sample, of the fecal microbiota from PD patients and controls [39, 42, 43, 45], most did find statistically significant differences in the beta-diversity, the community differences between the study groups [39, 41–44, 46, 47, 55]. Unsurprisingly, many of the studies found bacteria from the phyla Firmicutes and Bacteroidetes to dominate in the feces of both PD patients and controls [39, 46, 52]. From the phylum Bacteroidetes, the family Prevotellaceae, which has been implicated in the pathogenesis of IBD [56] was found to be altered in most of the studies: six studies found a dramatic decrease of Prevotellaceae in the feces of PD patients [43–48, 51, 55] with Scheperjans et al. even finding such a strong correlation between low abundance of Prevotellacea and PD phenotype that it could be used as a classifier with over 90% specificity [46]. Minato et al. found Prevotellacea to decrease in the feces of PD patients two years after the first sample collection [57]. Keshavarzian et al., however, found no such difference in the feces of PD patients and controls but did see a reduction by approximately 50% of Prevotellacea in the sigmoid mucosa of PD patients, although this did not reach statistical significance [39]. Additionally, Pereira et al. found a decrease of Prevotellaceae in the oral cavity of PD patients compared to healthy controls [41]. Interestingly, Qian et al. found a higher abundance of Paraprevotella, a member of the Prevotellaceae family, in PD patients compared to controls [42]. A study has found a five-fold decrease of Paraprevotella in the feces of multiple system atrophy (MSA) patients compared to healthy subjects [58]. Additionally, chronic exposure to rotenone resulted in a decrease of Paraprevotella in the feces of mice already one week post initiation of treatment [59]. In the case of Bifidobacteriaceae, the findings in different studies have been contradictory: Bedarf et al. reported a lower abundance of Bifidobacteriaceae in PD patients [44], Keshavarzian et al. found a lower abundance of Bifidobacteriaceae in both the mucosa and feces of PD patients compared to controls [39] whereas Hopfner et al., Li et al. and Lin et al. described the opposite [47, 49, 54]. Hill-Burns et al. also found an increased abundance of Bifidobacteriaceae in the feces of PD patients compared to controls but this difference disappeared once they accounted for medication, specifically the use of COMT inhibitors, as a confounding factor [50]. Minato et al., however, found that patients with lower counts of Bifidobacteriaceae had a more severe worsening of symptoms 2 years later compared to those with originally higher counts [57]. Mihaila et al. found an increased abundance of Bifidobacteriaceae in the saliva of PD patients compared to controls [60]. In a rotenone-induced mouse model of PD, Bifidobacteriaceae from the ceacal content and mucosa was reduced compared to control mice [61]. Enterobacteriacea were found to be more abundant in PD patients by four studies [39, 49, 51, 53], decreased in one [44] and unchanged in one [48]. Lactobacillaceae were found to be more abundant in PD patients’ stool in six studies [43, 46, 47, 49, 50, 53] and lower in two [42, 44]. Mihaila et al. found an increased abundance of Lactobacillaceae in the saliva of PD patients compared to controls [60]. Erysipelotrichaceae is another bacterial family which 3 studies found to be increased in PD stools [40, 42, 43] but 3 others found to be decreased or unchanged compared to healthy controls [39, 44, 49]. The butyrate producing Roseburia [39, 44, 50], Blautia [39, 44, 49, 50], and Faecalibacterium [39, [43–45], 49, 51] and Dorea [39, 44, 45] have been reported to be less abundant in PD patients’ stool. Interestingly, a study has shown that infection with *Clostridium difficile* results in dysbiosis characterized by an over-abundance of Lactobacillaceae and a decrease of Roseburia, Blautia, Faecalibacterium, and Dorea [32]. Akkermansia was consistently found to be more abundant in PD stool samples [39, 40, 43, 44, 50–52]. *Akkermansia muciniphila* is a recently identified mucin-dwelling species of the genus Akkermansia. *A. muciniphila* has been shown to mediate glucose tolerance via an IFNγ dependent mechanism and protect against obesity in mice [62]. Additionally, reduced levels of *A. muciniphila* in gut microbiota were reported for children with asthma [63]. A recent study showed that *A. mucniphila* can induce different T-cell responses based on the other species present in a given microbiota [64]. This can then explain why *A. muciniphila*, generally thought to mediate anti-inflammatory effects, has surprisingly been shown to be over-abundant in PD feces compared to controls. Finally, although most studies focused mainly on bacteria, a few studies reported an increase in methanogens of the archaeal domain in PD stools compared to controls [42, 44, 50, 51] and Bedarf et al. reported a decrease in virus counts in PD patients’ stools [44].

**Table 1:** Table summarizing key findings from stool microbiome studies in PD patients compared to healthy controls

		Qian et al. [42]	Lin et al. [43]	Bedarf et al. [44]	Petrov et al. [45]	Keshavarzian et al. [39]	Scheperjans et al. [46]	Hopfner et al. [47]	Hasegawa et al. [48]	Li et al. [49]	Hill-Burns et al. [50]	Unger et al. [51]	Heintz- Buschart et al. [40]	Li, F et al. [52]	Pietrucci et al. [53]	Lin, A et al. [54]
Alpha diversity		+*	+*	=	-*	+*	=	=		=					=	=
Beta diversity		*	*	*	*	*	*	*			*			*	*	*
**Family**	**Genus**															
Enterobacteriaceae				-		+			≈	+		+*			+*	
Pasteurellaceae				-						-	-*			+*		-*
Bifidobacteriaceae				-		-		+		+	+*					+*
	Bifidobacterium			-	+*	-			=		+*	+*				
Bacteroidaceae				-		+*		+		-	-*					
	Bacteroides			-	-*	+*					-*			-*		
Porphyromonadaceae			+*	-		+				-	+*			+*		
Prevotellaceae			-*	-*		=	-*	-		=		-*	-			
	Prevotella		-*	-*	-*	=			-				-	-*		
Rikenellaceae		+*	+*	+		+				-						
Lactobacillaceae		-*	+*	-			+*	+*		+	+*				+*	
	Lactobacillus	-*	+*	-	+*				+*		+*	-*				
Enterococcaceae	Enterococcus		+*	-					+	+*		-*				
Lachnospiraceae				-*		-*				-	-*			+*	-*	-*
Ruminococcaceae				-		+	+	-		-				+*		
	Faecalibacterium		-*	-	-*	-				-*	-*	-*				
Erysipelotrichaceae		+*	+*	-*		-				=			+			
Verrucomicrobiaceae			+*	+*		+*	+*	+		=	+*		+*	+*		
Akkermansiaceae	Akkermansia		+*	+*		+*					+*	+	+*	+*		

+ = higher in PD patients compared to controls; - = higher in controls compared to PD patients; * = *p* < 0.05

### Shortcomings of microbiome studies in PD research

With more fields recognizing the microbiome as a key player in diseased and healthy conditions, it should come as no surprise that the field of PD research too has focused on microbiota research in recent years. Unfortunately, it is still difficult to draw conclusions from the studies as most of them report contradictory findings. The wet lab techniques differed between the studies with most sequencing the hypervariable regions of the 16S rRNA gene but some testing for only specific bacterial taxa. Although 16S rRNA sequencing is a powerful tool in microbiome studies, it does have limitations: limited resolution, a dependence on databases chosen for species identification, reporting only on nucleic acid as compared to viable species, and relative results rather than absolute quantification of present species [65, 66]. Additionally, the time from sample collection to freezing varied greatly and storage of samples at room temperatures even for short periods of time are known to distort the relative abundances of bacterial taxa. The different studies also corrected for different confounding factors, such as medication, and were carried out in different geographical locations, factors known to affect microbiota composition. None of the studies provided information on antibiotic use in early life or previous *C. difficle* infections, which are known to have profound and long-lasting effects on gut microbiota. Additionally, only one study accounted for transit time as a confounding factor [50] although most of the studies reported PD patients as suffering from constipation and GI symptoms in higher frequency. Transit time has a clear effect on fecal consistency, which is known to have the highest impact on microbiota composition [36, 38]. Both *Akkermansia* and methanogens, here reported to be altered in PD patients’ stools, have been correlated to stool consistency [36]. A summary of the techniques and criteria employed by each study can be found in Table 2. Unfortunately, not all studies reported all findings, making it difficult to compare and perform meta-analytical studies among them. Finally, it is important to keep in mind that the majority of the studies focused on fecal composition, yet the GI tract is not homogenously colonized but rather consist of a multitude of niches [67, 68].

**Table 2:** Summary of the techniques and criteria employed by the different microbiome studies of PD patients compared to healthy controls

	Country of sample collection	*N* = PD patients	*N* = controls	Criteria for controls	Average disease duration	Exclusion criteria	Additional notes	DNA/RNA	Sequencing method	Sample handling
Qian et al. [42]	Shanghai, China	45	45	Spouses	5.7 years		All patients on medication	DNA	V3-V4 amplification; Illumina Miseq	Collected at home in the morning, shipped on ice
Lin et al. [43]	Taiwan	80	77	Age and sex-matched	7.5 years	IBD, antibiotics or prebiotics within past 3 months		DNA	V3-V4 amplification; Illumina Miseq	Samples immediately flash frozen with DNA stabilizer and stored at -80
Bedarf et al. [44]	Bonn, Germany	31	28	Only male participants, age-matched	Less than one year	Atypical or secondary PD, chronic inflmmatory GI symptoms including chronic constipation, use of laxatives or immunosuppressants within past 3 months	L-DOPA naive		Shotgun sequencing with Illumina Hiseq	
Petrov et al. [45]	Moscow, Russia	89	66					DNA	V3-V4 amplification; Illumina Miseq	
Keshavarzian et al. [39]	Chicago, USA	38	34		6.4 years	Atypical or secondary PD, use of antibiotics or prbiotics within 3 months, primary GI pathology, unstable neurology or psychiatric illness, low platelet count prolonged prothrombin time or history of bleeding		DNA	High-trhoughput amplicon sequencing of V4	
Scheperjans et al. [46]	Helsinki, Finland	72	72	Age and sex-matched	5 years (median)	Motor symptom onset before age 50 and having more than one relative or first-degree relative with PD, smoking, dementia, depression or psychosis, HIV, primary GI disease, endocrinological disease, alcohol abuse, pancreatic disease, bleeding disorders, infections, antibiotic use within last month, etc.		DNA	Pyrosequencing of V1-V3 regions	Collected at home, stored with DNA stabilizer; frozen at -80 within 3 days
Hopfner et al. [47]	Kiel, Germany	29	29		11 years	GI comorbidities and antibiotic use within last 3 months		DNA	V1-V2 amplification; Illumina Miseq	Collected at hospital or home, no preservation for up to 48 hours, then stored at -80
Hasegawa et al. [48]	Nagoya, Japan	45	35	Spouses	9,5 years	Atypical or secondary PD, use of antibiotics or prbiotics within 3 months, primary GI pathology, unstable neurology or psychiatric illness		RNA	RT-qPCR of 16S or 23S rRNA	Collected at hospital, stored with RNA stabilizer, stored at 4C
Li et al. [49]	Beijing, China	24	14	Age and sex-matched				DNA	V3-V5 amplification; Illumina Miseq	Collected at hospital or home, stored at -80C
Aho et al. [55]	Helsinki, Finland	64	64	Age and sex-matched	7.5 years (median Scherpejans et al. + 2.5)		Follow-up to Scherpejans et al. Same participants	DNA	V3-V4 amplification; Illumina Miseq	Collected at home, stored with DNA stabilizer; frozen at -80 within 3 days
Pereira et al. [41]	Helsinki, Finland	72, 69 (oral/nasal)	76, 67 (oral/nasal)	Age and sex-matched	5 (median)	Motor symptom onset before age 50 and having more than one relative or first-degree relative with PD, smoking, dementia, depression or psychosis, HIV, primary GI disease, endocrinological disease, alcohol abuse, pancreatic disease, bleeding disorders, infections, antibiotic use within last month, diseases of nose or mouth cavity, etc.		DNA	V3-V4 amplification; Illumina Miseq	Swabbing of oral and nasal cavities, on ice for 20 mins, stored at -80C
Hill-Burns et al. [50]	Seattle, Atlanta, and Albany; USA	197	130	54 pairs were spouses; rest unrelated	13.7 years			DNA	Illumina Miseq	Collected at home, shipped with postal services at ambient temperatures
Unger et al. [51]	Saar, Germany	34	34	Age-matched	8 years	Chronic or acute GI diseases, probiotic or antibiotic use within past 3 months	All patients on dopamine medication	DNA	RT-qPCR	Collected at home, sent to center, stored at -35C
Minato et al. [57]	Nagoya, Japan	36	78		11.5 years (2 years after Hasegawa et al.)		Follow-up to Hasegawa et al.	RNA	RT-qPCR of 16S or 23S rRNA	Collected at hospital, stored with RNA stabilizer, stored at 4C
Heintz- Buschart et al. [40]	Helsinki, Finland	76	10		7 years	Other neurological symptoms	Only 84 fecal samples were included in final analysis	DNA	V4 amplification; Illumina Hiseq	
Li, F et al. [52]	Jinzhou, China	10	72		6.2 years	Primary GI disease, alcohol abuse, pancreatic disease, B-hypovitaminosis, infection or antibiotic use within past month, sever gynecological prolapse, regular use of medication		DNA	Pyrosequencing of V1-V3 regions	Samples immediately flash frozen with DNA stabilizer and stored at -80
Pietrucci et al. [53]	Rome, Italy	80		Mostly spouses		Atypical or secondary PD, use of antibiotics or prbiotics within 3 months, primary GI pathology, unstable neurology or psychiatric illness, anamnesis, autoimmune or infectious diseases		DNA	V3-V4 amplification; Illumina Miseq	Collected at home, stored with DNA stabilizer stored at ambient temperatures
Lin A et al. [54]	Guangzhou, China	75	45	Spouses	4.5 years	Atypical or secondary PD, use of antibiotics or prbiotics within 3 months, primary GI pathology, unstable neurology or psychiatric illness		DNA	V4 amplification; Illumina Hiseq	Samples immediately flash frozen and stored at -80

All studies used the 16S rRNA gene for sequencing unless otherwise stated

### The microbiome in IBD

A study conducted on mouse models of colitis found Immunoglobulin A (IgA) coating to selectively identify pathology-driving bacteria. Genera from the orders Bacteroidales and Lactobacillus and family of Erysipelotrichaceae were particularly IgA-coated. Members of Clostridiales, Ruminococcaceae, Rikenellaceae, Roseburia, Faecalibacterium, and Blautia were identified from IBD patients as being selectively IgA-coated compared to healthy controls. The abundance of all these bacterial taxa have been shown to be altered in the stools of PD patients. Of interest is whether these taxa are IgA coated in PD patients too? In that same study, they also identified bacteria that were similarly IgA coated in IBD patients and healthy controls. Fecal transplantation of IgA-coated bacteria into mice did not result in pathology unless the mice were genetically susceptible to IBD [56]. This supports the interaction of genetics and environment in a dual-hit hypothesis, valid for IBD as well as PD.

### Host-microbiome interactions

The interactions between the host and microbiome are manifold, complex and bidirectional. It is now well established that gut bacteria can modulate the host immune response: polysaccharides produced by *B. fragilis* and *Bifidobacterium breve* can induce regulatory T-cells (Tregs) and stimulate the secretion of the anti-inflammatory cytokine IL-10 [68, 69]. Other bacteria induce inflammation, as has been well described in IBD [56, 70, 71]. The systemic effect of gut inflammation and its potential role in neurodegeneration has recently become an area of great interest. Some bacteria in the intestine also produce SCFAs, which serve as a main energy source for colonic epithelial cells and can also induce the generation of Tregs [71]. Butyrate, a SCFA, has been shown to exert regulatory functions on dendritic cell differentiation [72]. Although some of the studies found a reduction in butyrate-producing bacteria, generally thought of as anti-inflammatory strains, the exact effect of SCFAs is still under debate with some studies even reporting exacerbation of inflammation related to SCFAs [19]. In an MPTP-induced mouse model of Parkinsonism, fecal transplant resulted in a reduction of SCFAs accompanied by an amelioration of pathology [73]. On the other hand, miRNA produced by intestinal epithelial cells can enter bacteria and regulate gene transcription and growth [74]. The fecal miRNA patterns in PD patients have been shown to differ from healthy controls [75]. Bacterial lipopolysaccharide, in turn, has been shown to alter miRNA expression in macrophages leading to a cascade of inflammatory responses [76].

### Microbe-microbe interactions

Besides the effects mediated by the host on the microbiome, the constituents of the microbiome affect its composition too. Many bacteria, including commensals in our gut, possess quorum-sensing abilities: they can regulate and inhibit growth of other bacteria by secreting anti-microbials. Examples of quorum-sensing-capable bacteria are *E. coli, B. subtilis*, and species of *Streptococcus, Streptomyces and Lactobacillus*. Bacteria can also modulate the growth of other bacteria by competition for the same resources. The microbiome consists not only of bacteria though, and fungi, such as *Candida albicans* can also modulate the bacterial composition of the gut. *C. albicans* can co-aggregate and form mixed biofilms with for instance *Streptococci* and can compete with species such as *Pseudomonas aeruginosa* [77–79]. Additionally, although underreported, bacteriophages are among the most abundant microbes in our gut. In an experimental mouse model, infection with lytic bacteriophages had direct effects on susceptible bacterial populations, such as *E. coli*, and indirect effects on non-targeted bacteria through bacteria-bacteria interactions. The expansion of *A. muciniphila* was one of the indirect effects reported in that study [80].

### Conclusion and future perspectives

Microbiome studies generate a wealth of information that is difficult to interpret. Host-microbiome interactions will surely continue to hold interest in many research fields including that of neurodegenerative diseases. Establishing a causal relationship between microbiome dysbiosis and disease initiation or progression could open the door for biomarker and identify possible intervention targets. Although all of the patients included had idiopathic PD, it would be interesting to compare microbiota from PD patients with different familial mutations: do different forms of α-syn and phenotypic presentations of PD result in specific changes to the gut environment? Additionally, do different synucleinopathies share similar changes to the microbiota? There is no doubt that the field of microbiome studies holds great promise, however, as of now, fecal microbiota cannot be used as a biomarker for PD but perhaps inspiration could be drawn from the fields of obesity and IBD studies where machine learning has successfully been employed to aid in the identification of classifiers [22, 81, 82].

## Supplementary information

**Additional file 1: Table S1.** Compliation of the reported findings from stool microbiome studies comparing PD patients to healthy controls. + = higher in PD patients; - = higher in controls subjects; * = *p* < 0.05.

## Data Availability

All data generated or analyzed during this study are included in this published article and its supplementary information files.

## Abbreviations

(COMT): Catechol-O-methyltransferase
(DMNV): Dorsal motor nucleus of the vagus
(IBD): Inflammatory bowel disease
(LBs): Lewy bodies
(LNs): Lewy neurites
(MPTP): 1-metyl-4-fenyl-1,2,3,6-tetrahydropyridin
(PD): Parkinson’s Disease
(SCFAs): Short chain fatty acids
(Tregs): Regulatory T-cells
(α-syn): Alpha synuclein
A (IgA): Immunoglobulin
